# Comparative genomics of *Staphylococcus aureus* associated with subclinical and clinical bovine mastitis

**DOI:** 10.1371/journal.pone.0220804

**Published:** 2019-08-07

**Authors:** Lis S. Rocha, Danielle M. Silva, Mônica P. Silva, Pedro Marcus P. Vidigal, José Cleydson F. Silva, Simony T. Guerra, Márcio G. Ribeiro, Tiago Antônio de O. Mendes, Andréa de O. B. Ribon

**Affiliations:** 1 Departamento de Bioquímica e Biologia Molecular, Universidade Federal de Viçosa, Viçosa, Brazil; 2 Núcleo de Análise de Biomoléculas, Universidade Federal de Viçosa,Viçosa, Brazil; 3 Instituto Nacional de Ciência e Tecnologia em Interações Planta Praga/BIOAGRO, Universidade Federal de Viçosa, Viçosa, Brazil; 4 Departamento de Higiene Veterinária e Saúde Pública, Faculdade de Medicina Veterinária e Zootecnia, UNESP/Botucatu, Botucatu, Brazil; Mississippi State University, UNITED STATES

## Abstract

Many efforts have been made to understand the pathogenesis of bovine mastitis to reduce losses and promote animal welfare. *Staphylococcus aureus* may cause bovine clinical mastitis, but it is mainly associated with subclinical infection, which is usually persistent and can easily reoccur. Here, we conducted a comparative genomic analysis between strains of *S*. *aureus* causing subclinical infection (Sau170, 302, 1269, 1364), previously sequenced by our group, and two well-characterized strains causing clinical mastitis (N305 and RF122) to find differences that could be linked to mastitis outcome. A total of 146 virulence-associated genes were compared and no appreciable differences were found between the bacteria. However, several nonsynonymous single nucleotide polymorphisms (SNPs) were identified in genes present in the subclinical strains when compared to RF122 and N305, especially in genes encoding host immune evasion and surface proteins. The secreted and surface proteins predicted by *in silico* tools were compared through multidimensional scaling analysis (MDS), revealing a high degree of similarity among the strains. The comparison of orthologous genes by OrthoMCL identified a membrane transporter and a lipoprotein as exclusive of bacteria belonging to the subclinical and clinical groups, respectively. No hit was found in RF122 and N305 for the membrane transporter using BLAST algorithm. For the lipoprotein, sequences of Sau170, 302, 1269, and 1364 with identities between 68–73% were found in the MDS dataset. A conserved region found only in the lipoprotein genes of RF122 and N305 was used for primer design. Although the polymerase chain reaction (PCR) on field isolates of *S*. *aureus* did not validate the findings for the transporter, the lipoprotein was able to separate the clinical from the subclinical isolates. These results show that sequence variation among bovine *S*. *aureus*, and not only the presence/absence of virulence factors, is an important aspect to consider when comparing isolates causing different mastitis outcomes.

## Introduction

Bovine mastitis is the most common and expensive disease in dairy cattle worldwide. It results in milk contamination by bacteria and their toxins, along with a reduction of milk production and a withdrawl period due to the presence of antibiotic residues [1]. In addition, mastitis may also cause death or require the slaughter of chronically-infected animals.

Clinical and subclinical mastitis can occur in dairy herds, although a higher incidence of the latter scenario is predominant [2–5]. Subclinical mastitis is characterized by a lack of symptoms, with no apparent changes in the milk except for a drop in milk quality and quantity, which is not always detected by farmers. Due to its silent development, subclinical manifestations frequently evolve into chronic infections. *Staphylococcus aureus* is one of the most representative pathogenic bacteria causing bovine mastitis and is widely distributed in dairy cattle herds in several countries [6]. It has been assumed that the *S*. *aureus* strains associated with chronic infections are different from those that cause transient infections [7], and are more likely to be transmitted and prevail through the herd due to the absence of clinical signs [8,9]. Mastitis caused by *S*. *aureus* is usually persistent, resistant to conventional antimicrobials and can easily reoccur. Therefore, the only way to avoid dissemination to the entire herd is to segregate or eliminate the infected animals. Hence, the culling of animals at the subclinical stage is imperative to disease control.

Previous studies were conducted in order to associate the virulence factors (VF) present in *S*. *aureus* to the clinical outcome of bovine mastitis [10–13], aiming to distinguish virulent isolates from less harmful ones. For example, the gene *seg*, which encodes an enterotoxin, has been associated with a decreased likelihood of bacteria causing intramammary infections during lactation in dairy cows [14]. The comparison between *S*. *aureus* Newbould 305 (N305), a strain isolated from mild mastitis, and *S*. *aureus* RF122, associated with severe clinical mastitis, revealed differences that could be associated with disease severity [15]. Nevertheless, there is still much to uncover in terms of *S*. *aureus* pathogenesis, which is hampering effective strategies to combat bovine mastitis [16].

## Materials and methods

### Ethics statement

This study was approved by the Ethics Committee on Animal Use (CEUA) from the Faculdade de Medicina Veterinária e Zootecnia, UNESP/Botucatu, São Paulo, (protocol 136/2017) and the Universidade Federal de Viçosa, Viçosa, Minas Gerais (protocol 85/2014).

### Microorganisms and culture conditions

The bacteria used in this study were maintained on BHI agar (Brain Heart Infusion, BHI HiMedia, Mumbai, India) at 37°C, and stored in the long term in BHI containing 20% glycerol. Field isolates of *S*. *aureus* were used to validate some findings obtained by *in silico* analysis. Field isolates (308, 340, 403, 1001, 1311, 1315, 1323, 76, 216, 1439, 2555, 3909, 5T18-19, 9T18-16, 10T18-59, 10T18-68, 14T18-13, 22T18-52, and 22T17-54) were used to validate the *in silico* analysis and originated from animals suffering from subclinical or clinical mastitis as described below.

### Animals and diagnosis of clinical and subclinical mastitis

A convenience sample of cows diagnosed with clinical (n = 8 farms) or subclinical (n = 4 farms) mastitis by *S*. *aureus* was used in this study. The animals came from 12 farms located in the state of São Paulo (n = 3) and Minas Gerais (n = 9), central region of Brazil, where dairy farming is common, with similar conditions of nutrition, manegemant and facilities. Samples of milk from both clinical and subclinical cases of mastitis were collected from only one farm; although from different animals. All the animals were subjected to the routine strip cup test and the California Mastitis Test-CMT (scores 1+ to 3+) for the diagnosis of clinical and subclinical mastitis [19], respectively, diagnosed by veterinary assistance of farms. The diagnosis of clinical mastitis was based on macroscopic abnormalities in the milk (pus, lumps, and blood streaks), the presence of clinical signs of inflammation on the mammary gland (swelling, pain or congestion of mammary gland affected) and/or systemic signs of illness (inappetence, fever, tachycardia, tachypnea, decubitus and alterations of ruminal movements [19–20].

The isolates number 76, 216, 1439, 2555, 3909, 5T18-19, 9T18-16, 10T18-59, 10T18-68, 14T18-13, 22T18-52, and 22T17-54 were collected from cows presenting the clinical signs of mastitis from eight different farms. The isolates number 308, 340, 403, 1001, 1311, 1315, and 1323 came from cows suffering of subclinical mastitis (2+ or 3+ scores). These animals with subclinical mastitis were from different farms and had no visible signs of clinical mastitis along the lactation, during nine months of the herds were visited.

### Functional classification, comparative analysis and identification of orthologous proteins

The genomes of *S*. *aureus* 1269 (ST1), *S*. *aureus* 302 (ST126), *S*. *aureus* 170 (ST126), and *S*. *aureus* 1364 (ST126), associated with subclinical mastitis, were previously sequenced and deposited in NCBI as LNOO000000000, LNOR00000000, LNOQ00000000, and LNOP00000000, respectively [14]. Hereinafter, they will be referred to as Sau1269, Sau302, Sau170, and Sau1364. Two strains causing clinical infection, *S*. *aureus* RF122 (ST151) [21] and *S*. *aureus* N305 (ST115) [15], were used for comparative analysis; their genomes have been fully sequenced and made available in DDBJ/EMBL/GeneBank under the accession numbers NC_007622 and AKYW00000000, respectively. In order to avoid gene artefacts due to the use of different methods and programs for coding sequence predictions, Prodigal version 2.50 [22] was used for gene prediction in all analyzed genomes. The analysis of completeness of the genome assembly was carried out by BUSCO v3 software [23] using the Bacteria Dataset for the order Bacillales. High quality genomes had predicted values above 95%. BLAST searches (http://Blast.ncbi.nlm.nih.gov/) were used for the functional annotation of protein sets, allowing them to be grouped into Clusters of Orthologous Groups (COG) families [24]. The contigs were also submitted to automatic annotation in the RAST server (Rapid Annotation using Subsystem Technology), through searches for homology in the SEED databank [25], and automatically contrasted using a BLAST search with the already-annotated contigs of the strains *S*. *aureus* RF122 and *S*. *aureus* N305. The algorithm OrthoMCL, with an inflation index of 1.5 [26], was used to cluster the protein sequences into orthologous clusters. The software Bowtie2 version 2.2.8 [27] was used to align sequenced reads to the RF122 and N305 genomes in order to check misassembled and low coverage regions. The absence of a gene was considered an assembly artifact if the original high quality reads with phred quality above 20 were mapped with 100% gene coverage and conserved density when compared to the gene sequence in the reference genome.

### Evaluation of single nucleotide polymorphism (SNP) in virulence factors (VF)

The repertoire of VF present in the subclinical mastitis strains was defined based on those previously listed in the *S*. *aureus* RF122 genome [21], complemented by other virulence-associated genes [15, 28–34] and the VF Database (http://www.mgc.ac.cn/VFs/) (S1 Table). The FASTA sequences of the proteins were used in a protein BLAST search [35] against the genomes N305, RF122, Sau170, Sau302, Sau1269, and Sau1364. The protein was considered present (+) in the genome if the match presented a query coverage ≥65%, e-value ≤10^−10^ and sequence identity ≥ 30%. Manual curation and comparison to the literature were performed in order to verify whether the absence or presence of proteins in the genomes was authentic or caused by the thresholds applied in the BLAST search. Moreover, the Bowtie2 analysis was also applied to validate the presence or absence of genes using high quality reads in addition to the assembled genes to avoid false negative results due to assembly artifacts, such as genes located in breaks of contigs or collapses of repetitive regions that could hinder the identification by BLAST alignment.

The analysis of SNPs was performed using CLC Genomics Workbench version 8.5.4 (Qiagen) by mapping the reads of the sequenced strains *S*. *aureus* 170, 302, 1269, and 1364 onto the genome of the reference strains *S*. *aureus* RF122 (GenBank accession NC_007622) and *S*. *aureus* N305 (AKYW01.1). Despite their haploid genome, bacterial populations could show heterozygosity across strains generations. However, in the SNPs analysis, we aimed to identify only the polymorphisms which were fixed on the analyzed *S*. *aureus* strains, in comparison with the reference genomes and that would possibly be linked to mastitis outcome. In the SNPs analysis, the reads were initially trimmed for quality (Q20 score) and to eliminate ambiguous nucleotides, and then filtered for length by selecting sequences greater than 50 nt. Then, the selected reads of each strain were mapped onto reference genomes using a global alignment (mismatch cost, 2; insertion cost, 3; deletion cost, 3; length fraction, 1.0; similarity fraction, 0.90). The SNPs were predicted using the Basic Variant Detection tool, considering the settings for a monoploid genome and filtering the polymorphic loci sequenced with a minimum 20X coverage (ploidy, 1; minimum coverage, 20; minimum frequency, 100; minimum quality, 20). To ensure that all reads were the same nucleotide, the "minimum frequency parameter" [only variants that are present at least at the specified frequency (calculated as 'count'/'coverage')] of CLC Genomics Workbench was adjusted to 100%.

### Prediction of surface and secreted proteins

The protein sequences of *S*. *aureus* 170, 302, 1269, 1364, N305, and RF122, were used as the input for the identification of surface and secreted proteins, using the programs PSORTb v3.0 [36], TMHMM 2.0c [37], Phobius 1.01 [38], LipoP 1.0a [39], and SignalP 4.1 [40]. All programs were used with default parameters, and PSORTb and SignalP were also set to Gram-positive bacteria. The outputs of these softwares were combined, and the protein sequences that passed through all filters were selected. The selected sequences were aligned with Clustal-Omega 1.2.3 [41], and then used to create a distance matrix in which distances were expressed as the number of substitutions per 100 amino acids [42]. The distances of the matrix were used to build a multidimensional scaling (MDS) scattered plot, using the RStudio Version 1.0.136 and the package bios2mds (from BIOlogical Sequences to MultiDimensional Scaling) [43]. The proteins of *S*. *aureus* 170, 302, 1269, and 1364 (subclinical mastitis), were colored blue, whereas those from RF122 and N305 (clinical mastitis) were colored red. To identify differences between strains causing clinical or subclincal infections, the annotation of the 5111 proteins was retrieved through a batch BLAST analysis using BLASTp (e-value 10^−10^) against the protein database of *S*. *aureus* RF122. Then, the amino acids sequences of selected virulence factors were used in a multiple sequence alignment by the Muscle algorithm [44]. A pairwise nucleotide sequence identity matrix was generated using Sequence Demarcation Tool version 1.2 (SDT v1.2) Linux version [45] and the plot matrix was obtained using ggplot2 packages in the R software (https://cran.r-project.org/).

The sequences present in the cluster cl3700, exclusive of the genomes of clinical strains according to OrthoMCL, were also identified in the MDS plot. A BLASTp search was performed against the MDS dataset to find the best hit of the cl3700 sequences in the strains causing subclinical mastitis. Then, the CDS of six strains were aligned and primers complementary to regions of high variablitiy among the sequences were designed and used in PCR reactions. S1 Fig shows a flowchart of the analyses done to identify and validate cl3700.

### Polymerase chain reaction

DNA extraction of field isolates of *S*. *aureus* was performed with the PureLink Genomic DNA kit (Invitrogen), with the addition of lysozyme (20 μg.ml^-1^) (Ultrapure Lysozyme, J18645, Affymetrix/USB) in the initial step. The primer sequences, amplicon sizes and amplification conditions are summarized in Table 1. The primers that amplify the nuclease gene [46] were used to confirm the species identification of *S*. *aureus* isolates. The reaction mixtures consisted of 50 ng of total DNA, 1U of Taq DNA polymerase Cellco Biotec, 0.2 μM of each primer, 0.2 mM deoxynucleotide triphosphate mixture, 1X reaction buffer containing 2.0 mM MgCl_2_, extra 1.0 mM MgCl_2,_ and Milli-Q water to increase the reaction volume to a final volume of 25 μL. The extra 1 mM MgCl_2_ was excluded from the PCR reactions that contained the primers LipoP-F-CS/LipoP-R-C. Amplicons were analyzed by electrophoresis in 1X Tris-acetate-EDTA on a 1.0% agarose gel and images were visualized under UV light after staining with 2 mg.ml^-1^ ethidium bromide.

**Table 1:** Primers used to validate the results obtained *in silico*.

Primer	Sequence (5’-3’)	Target	Origin of isolate^a^	Expected amplicon size	Amplification conditions ^b^^,^^c^	Source
cl3316F	ACGCAAAACCCTTTACTAGT	Transporter protein (cl3316)	Subclinical mastitis	548 bp	Annealing: 55°C for 45s; Extension: 72°C for 45 s	This study
cl3316R	GCAACAACTAGTAGGAGTGA					
LipoP-F-CS	ACGCAAAACCCTTTACTAGT	Lipoprotein (cl3700)	Clinical mastitis	331 bp	Annealing: 55°C for 45s; Extension: 72°C for 45 s	This study
LipoP-R-C	GCAACAACTAGTAGGAGTGA					
LipoP-F-CS	GYTTTGCGAAAACGTTAGAYATGTA	Lipoprotein (cl3700)	Subclinical and clinical mastitis	582 bp	Annealing: 55°C for 45s; Extension: 72°C for 1min	This study
LipoP-R-CS	TGCCTTCATCATTAATTGGACCAATC					
au-F3	GYTTTGCGAAAACGTTAGAYATGTA	Thermo-nuclease	S. aureus	359 bp	^c^	Sasaki *et al* (2010) [46]
au-nucR	GGTAAAYTCAATGTYCTTATRTCC					

^a^Type of manifestation presented by the animal from which the bacteria were isolated.

^b^For all pair of primers except auR-F3 and auR-nucR: initial denaturation: 95.0°C for 5 min; 35 cycles of denaturation at 95.0°C for 45 s, annealing and extension as described in the table; final extension at 72.0°C for 10 min.

^c^ Conditions described by Sasaki *et al* (2010) [46]

## Results

### Functional classification and comparative genomic analysis

**Fig 1:**
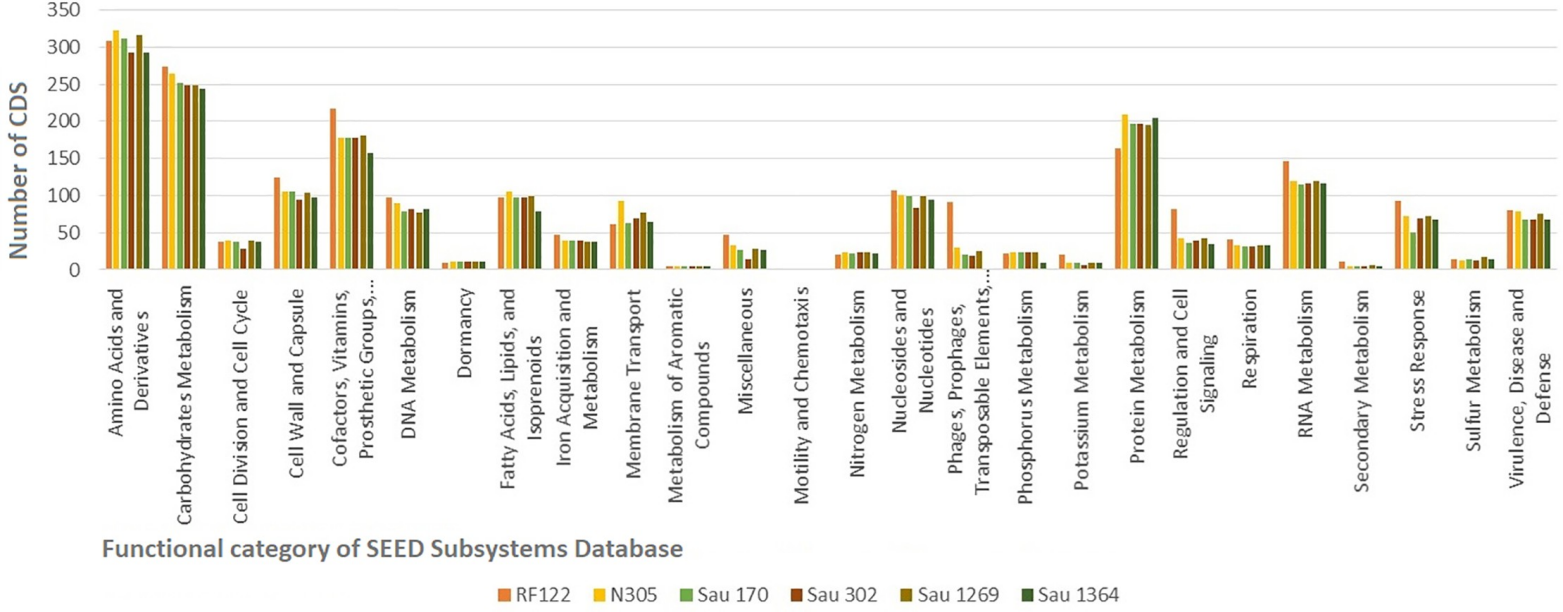
Functional classification of CDS from bovine *Staphylococcus aureus* sequenced genomes carried out using the SEED database. The graph represents an average of CDS distributions for the genomes of the strains *S*. *aureus* 170, 302, 1269, and 1364, isolated from animals suffering from subclinical mastitis, and the strains *S*. *aureus* N305 and *S*. *aureus* RF122, isolated from cases of clinical mastitis.

The BUSCO analysis confirmed the completeness of the genome assemblies with all strains presenting more than 96% conservation of single-copy orthologs for the order Bacillales. Approximately 77% of the proteins deduced from the genomes of the four sequenced subclinical strains were classified into COG families, and a similar distribution of the amount of proteins within the categories was seen. On average, 10% of the proteins of the sequenced genomes had unknown functions. Among the categories with the highest abundance, we identified proteins related to amino acids and derivatives, protein translation, and carbohydrate metabolism. Functional annotation of reads was also done using the SEED Subsystems Database (Fig 1) and 55% of the coding sequences (CDS) could be categorized, among which 5% were classified as hypothetical. Again, CDS were identified as belonging mainly to the functional category of amino acids and derivatives (16%), carbohydrates (13%), and protein metabolism (10%). Sau170, Sau302, and Sau1364 had 68 CDS assigned to the virulence, disease, and defense subsystem, while Sau1269 had 76. In Sau1364, only one sequence belonged to the phages, prophages, transposable elements, and plasmids subsystem compared to the 19 or more genes found in the same subsystem in the other genomes. Some categories were more frequently represented in *S*. *aureus* RF122 compared to the other strains, like phages, prophages, transposable elements, plasmids, and regulation and cell signaling.

### Genomic analysis of virulence differences

S1 Table shows the virulence-associated genes present or absent in the analyzed genomes, according to the BLAST thresholds used in this study. A total of 146 genes coding for VF were found, including toxins, exoenzymes, adhesion and cell wall anchored surface proteins, proteins related to host immune evasion, biofilm production, regulatory, and miscellaneous proteins. Toxin encoding genes were differently distributed among the genomes, which harbored the majority of the regulatory genes that were investigated. A total of 82 VF were present in all six genomes, while three were absent in all of them: chemotaxis-inhibiting protein *(chp*), collagen adhesin (*cna*), and staphylokinase (*sak*). The exfoliative toxin A gene (*eta*), known for causing staphylococcal scalded skin syndrome, and the transcriptional Repressor SaPI gene (*stl*), which controls the retention of the *Staphylococcus aureus* pathogenicity islands (SaPIs) in the host chromosome, were only found in the genomes of strains associated with clinical mastitis.

Seven genes were absent in all genomes of the strains causing subclinical infection: bovine variant of enterotoxin C (*sec-bov*), enterotoxin t (*set*), streptolysin S-associated protein *sagB* homolog, streptolysin-associated protein *sagD* homolog, toxic shock syndrome toxin 1 (*tst*), transcriptional repressor SaPI (*stl*), serine-rich adhesin for platelet (*sasA*), and capsular polysaccharide biosynthesis protein Cap5I (S1 Table). Some of them, however, were found in RF122 (*sec-bov*, *set*, *sagB*, *sagD*, *tst*) or N305 (*sasA*, *cap5I*). Comparatively, some genes were present in Sau170, Sau302, Sau1269, Sau1364, and N305 but were absent in RF122 (*sasC*, *sasG*, *sasK*, *sasB*, *fnbB*). The genes coding for leukocidin chain lukM precursor (*lukM*) and Panton-Valentine leukocidin (*pvl*), were found in all strains but N305. Among the strains causing subclinical infection, Sau1269 had the largest number of virulence-associated genes (123), followed by Sau170 (105), Sau1364 (103), and Sau302 (104) (S1 Table). The strain Sau1269 also presented more genes related to toxins (29/37) compared to strains Sau170 (15/37), Sau302 (16/37), and Sau1364 (14/37). Seventeen regulatory proteins were found in Sau1269 that also had 23 of the 28 genes coding proteins involved in host immune evasion. Sau170 had more genes coding for exoenzymes (13/15), adhesion and cell wall anchored surface proteins (20/25).

**Fig 2:**
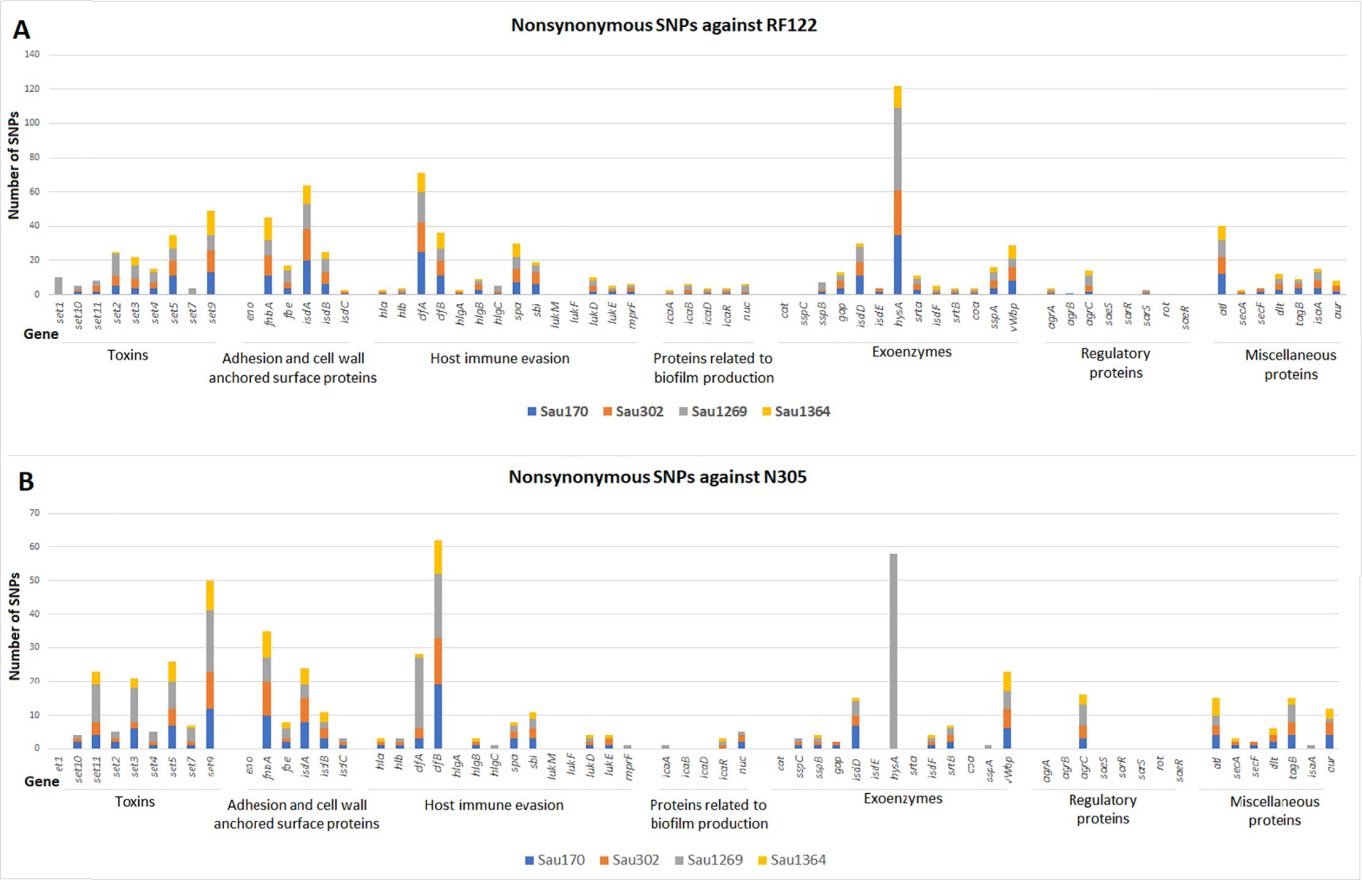
Nonsynonymous SNPs in virulence-associated genes present in the genomes of the subclinical strains. (A) SNPs mapped onto the genome of the clinical reference strain *S*. *aureus* RF122. (B) SNPs mapped onto the genome of the clinical strain *S*. *aureus* Newbould 305. The genes were grouped into their respective functional categories. Each bar represents a gene, and each color on the bars represents the number of SNPs for the gene that the corresponding genome strain displays when mapped onto the reference strain.

We used all genes coding for VF that were common to the two clinical genomes and to the six subclinical genomes for SNPs analysis (Fig 2, S2 Fig). The number of SNPs found in the CDSs of the genomes of the strains associated with subclinical infection was mapped onto the reference genomes of RF122 and N305. In these genes, a total of 32 to 36% of the SNPs was nonsynonymous, while the majority of them were synonymous. The majority of nonsynonymous mutations occurred among amino acids of the same class, conserving the physico-chemical properties of the residues. Although the total number of SNPs was different for each *S*. *aureus* strain, there was a similar distribution of the types of SNPs among them.

In general, more SNPs were found when the genomes of the subclinical strains were mapped onto RF122. Only the genes *clfB* and *set11* displayed more SNPs when mapped onto N305 than when mapped onto RF122. When the number of SNPs for both RF122 and N305 was summed up for each gene, the greatest numbers of nonsynonymous SNPs (180, 99, 98, and 98) were seen in *hysA*, coding for hyaluronate lyase, in the enterotoxin gene *set9*, and in the adhesin genes *clfA* and *clfB* (Fig 2). Similarly, the greatest numbers of total SNPs (329, 281, 221, and 217) were seen in *hysA*, *clfB*, *isdA*, coding for the iron-regulated surface determinant protein A, and *clfA* (S2 Fig). No SNPs were found in the genes coding for Leukocidin chain lukM precursor and Leukocidin F subunit, both related to host immune evasion, and in the gene of the response regulator SaeR.

### Prediction of surface proteins present in the genomes of the bovine *S*. *aureus* strains and validation of the multidimensional scaling (MDS) analysis

The programs PSORTb, TMHMM, Phobius, LipoP, and SignalP predicted a different number of transmembrane, surface, and secreted proteins for each of the six analyzed genomes. After the combination of outputs and the elimination of redundant proteins, a total of 922 proteins was predicted for N305, 971 for RF122, 947 for Sau170, 962 for Sau302, 984 for Sau1269, and 954 for Sau1364 (Table 2). TMHMM and Phobius predicted the greatest number of proteins, compared to the other programs. Most of the protein sequences were highly conserved among the genomes.

**Table 2:** Output of the programs used for the prediction of surface and secreted proteins of the studied *Staphylococcus aureus* strains.

Strain	LipoP	Phobius	PSORTb	SignalP	TMHMM	Total	Total^1^
*S*. *aureus* RF122	338	653	125	157	698	1971	971
*S*. *aureus* N305	321	666	160	151	689	1987	922
*S*. *aureus* 170	325	624	137	146	671	1903	947
*S*. *aureus* 302	323	637	145	136	673	1914	962
*S*. *aureus* 1269	356	654	130	161	688	1989	984
*S*. *aureus* 1364	317	633	146	139	670	1905	954

^1^ Total number of predicted secreted proteins without redundancy.

**Fig 3:**
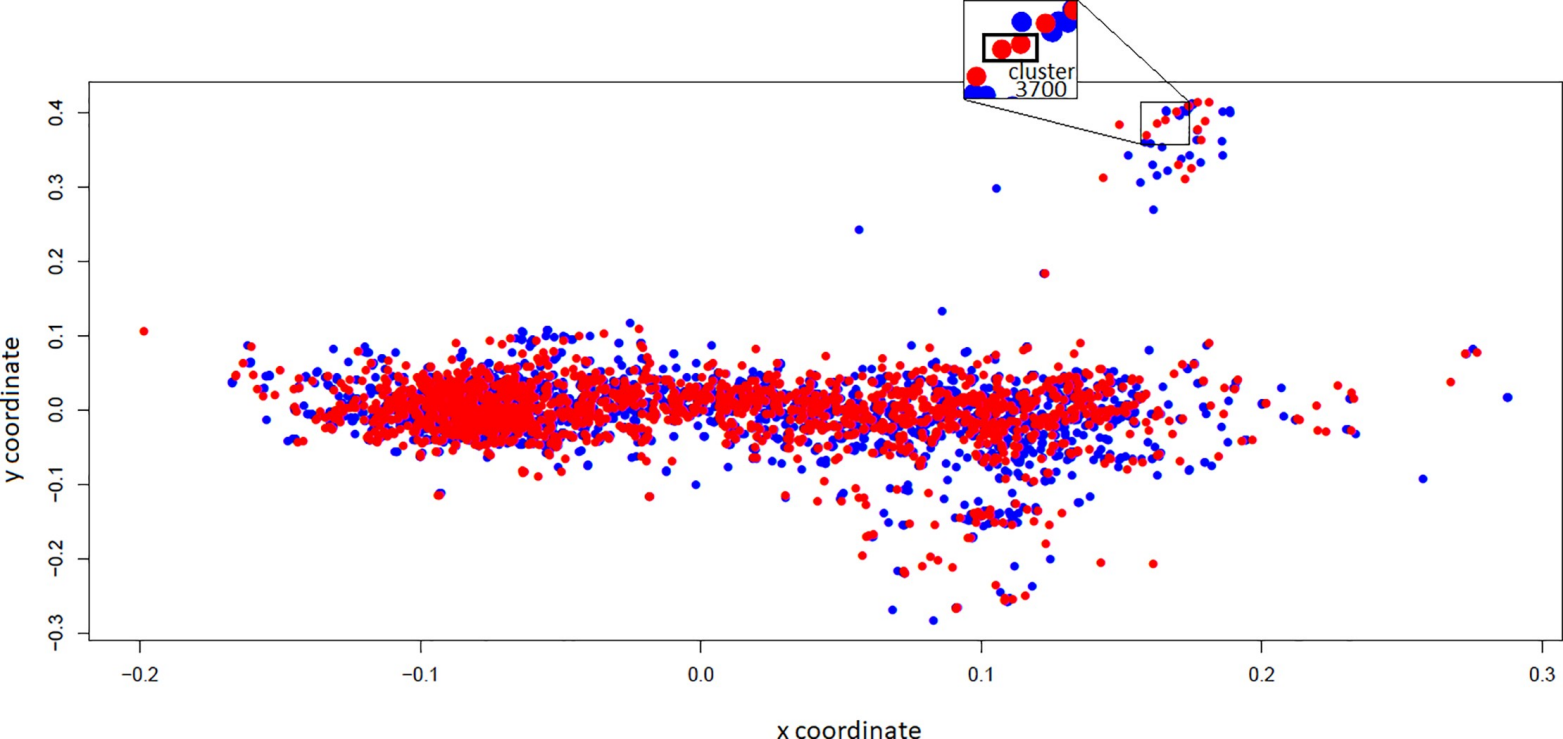
MDS plot of transmembrane, surface and secreted proteins generated from the proteins of bovine *Staphylococcus aureus* strains. The genomes of *S*. *aureus* 170, 302, 1269, and 1364 are in blue and the genomes of *S*. *aureus* N305 and RF122 are in red. Closely located dots represent more identical protein sequences. In the upper left and upper right, the clusters cl3009 and cl3700, respectively, are zoomed-in. Overlapped blue dots can be seen, corresponding to a protein that is highly conserved in the genomes of the strains isolated from cows with subclinical mastitis.

**Fig 4:**
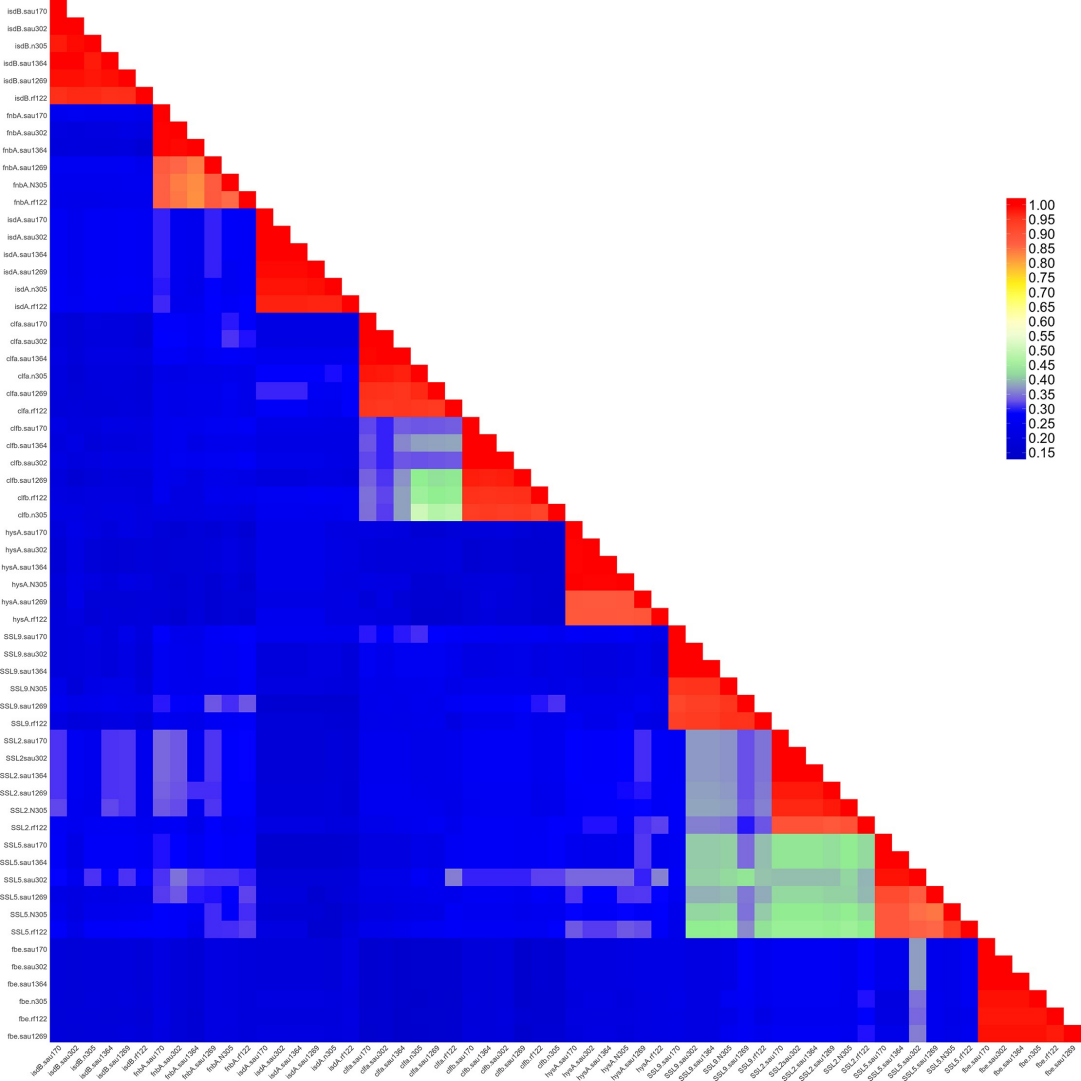
Pairwise identity matrix generated by alignment of selected virulence factors of bovine *Staphylococcus aureus* strains. Each colored cell represents the percentage of identity between two amino acid sequences that intersect in the cell, ranging from red (lowest identity) to blue (highest identity).

Following the alignment of the FASTA sequences of the proteins and the creation of a distance matrix, a MDS plot was built (Fig 3). The results showed no separation between the strains. In order to find putative differences among strains, the proteins analyzed in the MDS were annotated (S2 Table). Amino acid sequences of some proteins involved in adhesion and iron acquisition, and some secreted proteins were selected and used to generate an identity matrix (Fig 4, S3 Table), Overall, the orthologous proteins showed high identity among the strains. Based on identities, there was no separation between groups of strains associated with subclinical or clinical mastitis. In Sau170, 302, and 1364, six out of the ten orthologous proteins had 100% identity. Sequence identity of FnbpA ranged from 82.2% to 99.8%. 42.3% - 50% of identity was seen between ClfB and ClfA of Sau RF122, N305, and 1269. Ssl5 had around 40% identity with Ssl2 regardeless of the strain.

### Identification of orthologous proteins

The analysis of predicted proteins by OrthoMCL, an algorithm that allows ortholog group identification, retrieved several clusters likely to be exclusive in the genomes of the strains causing subclinical mastitis (S4 Table). However, manual curation through the alignment of high-quality short reads with phred higher than 30 to the RF122 and N305 genomes, using the program Bowtie 2, showed that this exclusivity was only real for nine clusters. In fact, the lack of correspondence of sequences between the strains was mainly caused by artefacts of genome assemblies, since the reads aligned and covered a corresponding gene in the genomes of strains causing clinical mastitis and, therefore, could not be considered exclusive. The cluster (cl3316) had sequences homologous to members of the major facilitator superfamily (MFS), the largest group of secondary active membrane transporters. Given that MFS are surface proteins, the sequences of cl3316 were used in a BLAST search to find the corresponding sequences of RF122 and N305 in the MDS dataset. No hit was found despite the presence of other MFS sequences in the clinical strains (S2 Table). Therefore, to validate the differences found *in silico*, primers were designed and used in PCR (see below).

OrthMCL also revealed 60 clusters exclusively present in the genomes of the clinical strains (S4 Table). Again, manual curation showed that most of them were assembly artifacts. Among the four candidates that were left, there was a cluster (cl3700) comprised of two sequences identified as a tandem-type lipoprotein. As lipoproteins are surface proteins, we used the MDS dataset to find sequences present in Sau170, 302, 1269, and 1364 with similarity to cl3700. The search retrieved only one hit from each strain. CDS alignment revealed a conserved region found in the sequences of the strains RF122 and N305 that was not present in the other strains (S3 Fig) and that was used to validate the differences among them by PCR.

### Polymerase chain reaction

To investigate whether the results found in the multi-sample comparison would be able to discriminate between field isolates causing subclinical and clinical mastits, we performed PCRs with different sets of primers (Table 1). Total DNA from the four strains that had their genome sequenced were also tested. An amplicon of the expected size was seen when total DNA was amplified with primers for the *nuc* gene, confirming the isolates as *S*. *aureus* (S4 Fig).

Experimental validation of *in silico*-derived results of the cl3316 showed a 548 bp-amplicon in 22/24 samples (S5 Fig). Therefore, the PCR results did not confirm the OrthoMCL findings. Two sets of primers (Table 1) were used to differentially amplify the lipoprotein (cluster3700): LipoP-F-CS / Lipo-R-CS, based on a region conserved in the lipoprotein gene of the six sequenced genomes, and LipoP-F-CS / LipoP-R-C, based on a region conserved only in the genomes of the clinical strains RF122 and N305 (S3 Fig). A 582 bp fragment, product of the set of primers LipoP-F-CS/LipoP-R-CS, was amplified in 22/24 field isolates, regardless of the type of manifestation (S6A Fig). On the other hand, a 331 bp fragment, product of the set of primers LipoP-F-CS/LipoP-R-C, was detected only in the isolates originating from animals with clinical manifestations of mastitis, whereas DNA from bacteria isolated from subclinical mastitis was not amplified (S6B Fig). Table 3 summarizes the PCR results for the sets of primers for each isolate tested.

**Table 3:** Summary of PCR results using the primers described in Table 1.

*S*. *aureus* isolate	Type of manifestation	nucAur	cl3316F/R	LipoP-F-CS/LipoP-R-C	LipoP-F-CS/LipoP-R-CS
170	Subclinical	+	+	-	+
302	Subclinical	+	+	-	+
1269	Subclinical	+	+	-	+
1364	Subclinical	+	+	-	+
308	Subclinical	+	+	-	+
340	Subclinical	+	+	-	-
403	Subclinical	+	+	-	+
1001	Subclinical	+	+	-	+
1311	Subclinical	+	+	-	+
1315	Subclinical	+	+	-	+
1323	Subclinical	+	+	-	+
76	Clinical	+	+	-	+
216	Clinical	+	-	+	+
1439	Clinical	+	+	+	+
3909	Clinical	+	+	-	+
2555	Clinical	+	+	+	+
5T18-19	Clinical	+	+	+	-
9T18-16	Clinical	+	+	+	+
10T18-59	Clinical	+	+	+	+
10T18-68	Clinical	+	+	+	+
14T18-13	Clinical	+	+	+	+
22T18-52	Clinical	+	+	+	+
22T17-54	Clinical	+	+	+	-
ATCC 29213*	*Human	+	-	+	+

*ATCC 29213 is a strain isolated from a human infection and was used as a control for the primer nucAur, specific for *S*. *aureus*.

## Discussion

Our group sequenced four genomes of *S*. *aureus* isolated from strains causing subclinical mastitis [17]. In this study, a comparative analysis was conducted with the genomes of *S*. *aureus* RF122 and N305, two well-characterized strains isolated from cows with clinical mastitis, in order to find bacterial factors that could be linked to disease outcome.

This is the first genomic analysis of a bovine *S*. *aureus* assigned to ST126; this type is prevalent in Brazilian herds [47], which has been claimed to be a more significant ST to define methods for controlling *S*. *aureus* infections due to its site specificity [18]. More than 70% of the genes could be grouped into specific COG categories, similarly as described for *S*. *aureus* RF122 [21] and N305 [15]. Moreover, BUSCO results showed extensive conservation of single-copy orthologs in the Bacillales, confirming the completeness of the assembled genomes. Regardless of the database used (COG or SEED), the categorization of the genes and their distribution in subsystems were similar among the sequenced genomes. However, *S*. *aureus* RF122 had more sequences related to stress response, regulation and cell signaling, potassium metabolism, cofactors and vitamins, plasmids, transposable elements, and prophages, which could confer the ability of the bacteria to cause severe mastitis. The phages, prophages, transposable elements, and plasmids subsystem of all subclinical strains except Sau1364 had a similar number of genes. This could be explained by the genetic variability among the strains and differences in the genomic regions covered by the assembled contigs. In *S*. *aureus* of human origin, great variability between strains has been reported in the mobile genomic elements; this could also be true for bovine strains. For example, four integrated prophages have been identified in *S*. *aureus* Newman and only one has been identified in *S*. *aureus* COL.

Here, we expanded the repertoire of VF previously described for bovine strains of *S*. *aureus* [15, 11], and looked for other determinants reported for human strains [28–34]. The gene content was quite similar among strains, and associations between mastitis outcome and VF were difficult to make. However, there were some exceptions. Genes that code for enterotoxins were mostly present in RF122 and Sau1269, such as the enterotoxins A, B, G, I, J, K, L, M, N and the enterotoxin-like proteins U and V. Enterotoxins are considered superantigens due to their stimulation of T-cell proliferation [48] and may impact disease severity in a rabbit model [49]. However, Sau1269 was isolated from an animal diagnosed with subclinical infection, suggesting that host factors, in addition to toxin presence, affects mastitis outcome. Also, there could be regulatory differences between RF122 and Sau1269 but the expression of enterotoxin genes was not investigated.

The exfoliative toxin A gene (*eta*) and the and the transcriptional Repressor SaPI gene (*stl*) were only present in RF122 and N305. Other genes such as the ones coding for enterotoxin t (*set*), toxic shock syndrome toxin 1 (*tst*), and streptolysin S-associated protein SagB/D homologs (*sagB* and *sagD*) were exclusive to RF122 and could have a role in the severe mastitis caused by this strain. Capra *et al*. also sequenced the genomes of six *S*. *aureus* strains (ST398 and ST8), that caused subclinical mastitis, which were divided into two groups (low and high within herd prevalence) [50]. Contrary to their findings, the gene that codes for the collagen binding protein (*cna*) was not found in the strains causing subclinical mastitis. Additionally, the genes encoding clumping factor protein (*clfB*), fibronectin binding protein (*fnbA*), and leukotoxin D and E (*lukD* and *lukE*) were present in all strains, including N305 and RF122.

A total of 933 and 565 nonsynonymous SNPs was found when the virulence genes were mapped onto the RF122 and N305 genomes, respectively, although the majority were synonymous SNPs, as observed in isolates of the same species [51–52]. Given that these strains belong to different clonal complexes (CCs), most of non-synonymous mutations were probably removed through purifying selection. However, non-synonymous mutations that probably have been fixed in the CCs are important targets to discriminate each complex, as shown recently for Group B *Streptococcus* [53]. In this pathogen, genes with pivotal roles during the infection or colonization process have a significant mutational signature exclusively in strains of hypervirulent CC 17, when compared to other human-associated CCs.

Overall, there were more SNPs when genes present in the subclinical strains were compared to those of RF122, especially genes encoding host immune evasion (*spa*, *clfA*, *sbi—*120 SNPs) and surface proteins (*fnbA*, *isdA—*109 SNPs). On the other hand, the number of SNPs in these genes was 47 and 59, respectively, when the comparison was done with N305. Fibronectin binding proteins, such as FnbA and FnbB, are important for adhesion and invasion into bovine mammary gland cells [54–55]. Genetic variations in *fnbA* and *clfA* impaired the detection of *S*. *aureus* by the latex agglutination test, probably due to the reduced ligation of adhesins to fibrinogen molecules coupled to the latex particles [56]. Since higher adhesion is associated with higher invasion in mammary epithelial cells, the noted allelic variations might lead to phenotypic differences between strains, which could impact disease progression. Further work testing the effect of each SNP on toxicity and virulence aspects should be performed to confirm these propositions.

Routine diagnosis of bovine *S*. *aureus* is based on bacterial culturing. There are many reports describing the application of immunoassays [57] and mass spectrometry [58] to improve and hasten the diagnosis. However, other issues should be tackled, such as differentiation between sporadic and contagious strains [12] and between strains associated with specific mastitis outcomes. To date, studies have failed to find robust bacterial markers for bovine mastitis based on the presence or absence of VF. Here, we showed a high similarity of gene content among the genomes of six bovine strains, which may help explain the difficulties in associating bacterial factors to disease outcome; whether this is due to the small number of genomes that have been sequenced so far is yet to be shown.

Bioinformatics showed substantial similarities in the set of surface and secreted proteins of the bovine strains. Although the *in silico* analysis described the members of cl3316 as exclusive of the strains associated with subclinical mastitis, the findings were not validated in experimental analysis. It is possible that the primers designed to amplify cl3316 were also complementary to other MFS sequences present in RF122 and N305 given that MFS comprises 74 families of proteins involved in drug efflux mechanisms [59].

This work shifted the focus to sequence variation, like that found in the lipoprotein cl3700, which may determine the different outcomes of mastitis. Lipoproteins are anchored to membrane lipids, with an important role in immune activation through Toll-like receptor 2 [60], which ultimately cause the activation of NF-κB and inducement of proinflammatory cytokines [61]. In addition, the deletion of the νSaα specific lipoprotein-like cluster (*lpl*) of *S*. *aureus* USA300 significantly decreased invasiveness and the expression of TNF-α and IL-6 in human cell lines [62]. Differences in amino acid composition can affect folding or function, which could ultimately cause less activation or deceive the immune system of the host, and interfere in the clinical symptoms presented by the mastitic cows. Therefore, the noted variation in the lipoprotein cl3700 sequences may influence protein function and further guarantee an advantage for bacterial survival in the host, which manifests as a subclinical and persistent infection.

Although analysis has to be expanded to accommodate more field isolates, preliminary results show the potential of lipoprotein cl3700 to differentiate between the two groups of isolates collected from animals suffering from clinical or subclincal mastitis.

## Conclusions

This work reveals a high gene content similarity in the genomes of strains causing clinical or subclinical mastitis. However, a lipoprotein (cl3700) demonstrated a higher potential to distinguish the two groups of bacteria, revealing that sequence variation among bovine *S*. *aureus*, and not only the presence/absence of virulence factors, is an important aspect to consider when comparing field isolates causing different mastitis outcomes. The several SNPs detected on VF might confer advantages to the subclinical strains to successfully evade the immune system without triggering immune responses, therefore facilitating the establishment of chronic and silent infections.

## Supporting information

S1 Fig Pipeline describing the methodology used to find and test the lipoprotein cl3700. Red boxes and blue boxes are related to properties of the genomes of the clinical and subclinical strains, respectively, while methodologic steps were collored in green.
(TIF)

S2 Fig Total SNPs in virulence-associated genes present in the genomes of the subclinical strains. (A) SNPs mapped onto the genome of the clinical reference strain *S*. *aureus* RF122. (B) SNPs mapped onto the genome of the clinical strain *S*. *aureus* Newbould 305 (B). The genes were grouped into their respective functional categories. Each bar represents a gene, and each color in the bars represents the number of SNPs for the gene that the corresponding genome strain displays when mapped onto the reference strain.
(TIFF)

S3 Fig *In silico* analyses of the lipoprotein cl3700. At the top, the percent identity matrix created with Clustal 2.1 of the lipoprotein, built with the amino acid sequence for the genomes of *S*. *aureus* strains Sau170, Sau302, Sau1269, Sau1364, RF122, and N305. The region conserved in RF122 and N305 is boxed in red. At the bottom, a multiple sequence alignment of the lipoprotein, also created with Clustal Omega, and the design of the sets of primers LipoP-F-CS/LipoP-R-C and LipoP-F-CS/LipoP-R-CS over the regions conserved among the clinical and subclinical strains (LipoP-F-CS and LipoP-R-CS) and over the region conserved between the clinical strains only (LipoP-R-C), all boxed in yellow. The red boxes correspond to the regions conserved only in the clinical strains, while the blue boxes correspond to the regions conserved in the subclinical strains. Highly conserved regions are boxed in green.
(TIF)

S4 Fig Amplification of the nuclease gene of *Staphylococcus aureus* of bovine origin. The PCR reaction was performed with the DNA extracted from the isolates *S*. *aureus* 170 (1), 302 (2), 1269 (3), 1364 (4), 308 (5), 340 (6), 403 (7), 1001 (8), 1311 (9), 1315 (10), 1323 (11), strain ATCC 29213 (12) and the DNA extracted from the isolates *S*. *aureus* 76 (13), 216 (14), 1439 (15), 3909 (16), 2555 (17), 5T18-19 (18), 9T18-16 (19), 10T18-59 (20), 10T18-68 (21), 14T18-13 (22), 22T18-52 (23), 22T17-54 (24). Water was used as a negative control (25). Promega 1 kb DNA Ladder was used as a molecular weight marker.
(TIFF)

S5 Fig Amplification of the gene coding the transporter protein cl3316 of *Staphylococcus aureus* isolates of bovine origin. The PCR reaction was performed with total DNA extracted from subclinical mastitis isolates *S*. *aureus* 170 (1), 302 (2), 1269 (3), 1364 (4), 308 (5), 340 (6), 403 (7), 1001 (8), 1311 (9), 1315 (10), 1323 (11) and from the clinical mastitis isolates *S*. *aureus* 76 (13), 216 (14), 1439 (15), 3909 (16), 2555 (17), 5T18-19 (18), 9T18-16 (19), 10T18-59 (20), 10T18-68 (21), 14T18-13 (22), 22T18-52 (23), 22T17-54 (24). Total DNA from *S*. *aureus* ATCC 29213 (12). Water was used as a negative control (25). Promega 1 kb DNA Ladder was used as a molecular weight marker.
(TIFF)

S6 Fig Amplification of a lipoprotein gene of *Staphylococcus aureus* isolates of bovine origin. At the top (A), the primers LipoP-F-CS and LipoP-R-CS were used in a PCR reaction lewith DNA extracted from the subclinical mastitis isolates *S*. *aureus* 170 (1), 302 (2), 1269 (3), 1364 (4), 308 (5), 340 (6), 403 (7), 1001 (8), 1311 (9), 1315 (10), 1323 (11) and from the clinical mastits isolates *S*. *aureus* 76 (13), 216 (14), 1439 (15), 3909 (16), 2555 (17), 5T18-19 (18), 9T18-16 (19), 10T18-59 (20), 10T18-68 (21), 14T18-13 (22), 22T18-52 (23), 22T17-54 (24). At the bottom (B), the primers LipoP-F-CS and LipoP-R-C were used to amplify the same DNA Total DNA from *S*. *aureus* ATCC 29213 (12). Water was used as a negative control (25). Promega 1 kb DNA Ladder was used as a molecular weight marker.
(TIFF)

S1 Table Presence (+) or absence (-) of virulence-associated genes in the analyzed genomes of bovine *Staphylococcus aureus*. (XLSX)

S2 Table Annotation of proteins present in the MDS plot through a batch BLASTp against *Staphylococcus aureus* RF122. (XLS)

S3 Table Identity matrix of secreted proteins and proteins involved in adhesion and iron acquisition encoded by the subclinical and clinical genomes. (XLSX)

S4 Table Manual curation of the orthoMCL clusters that were possibly exclusive to the genomes of *Staphylococcus aureus* subclinical strains (Tab A) or of the clinical strains (Tab B). (XLSX)
