# Supplementary figures and images for: Comparative genomics of Staphylococcus aureus associated with subclinical and clinical bovine mastitis
Source: PLoS One. 2019 Aug 7;14(8):e0220804. doi: 10.1371/journal.pone.0220804 (PMC6685620; doi:10.1371/journal.pone.0220804)

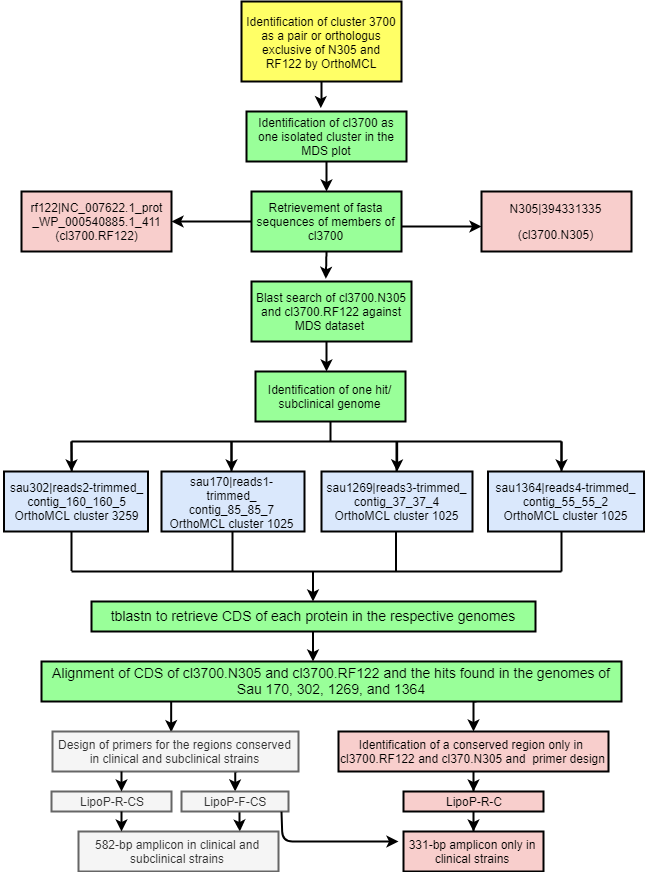

Supplement: S1 Fig — Red boxes and blue boxes are related to properties of the genomes of the clinical and subclinical strains, respectively, while methodologic steps were collored in green. (TIF) [file pone.0220804.s001.tif]

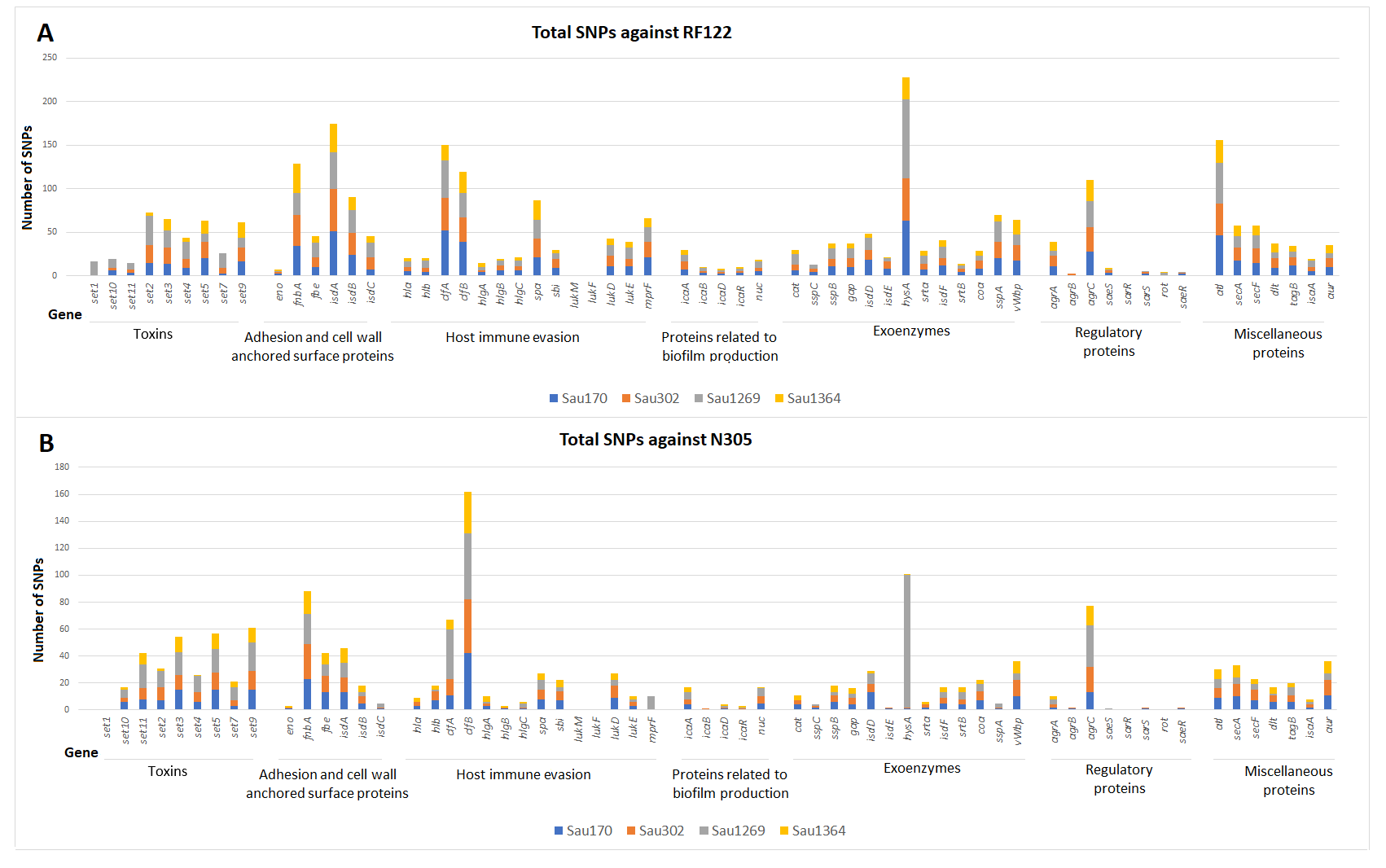

Supplement: S2 Fig — (A) SNPs mapped onto the genome of the clinical reference strain S. aureus RF122. (B) SNPs mapped onto the genome of the clinical strain S. aureus Newbould 305 (B). The genes were grouped into their respective functional categories. Each bar represents a gene, and each color in the bars represents the number of SNPs for the gene that the corresponding genome strain displays when mapped onto the reference strain. (TIFF) [file pone.0220804.s002.tiff]

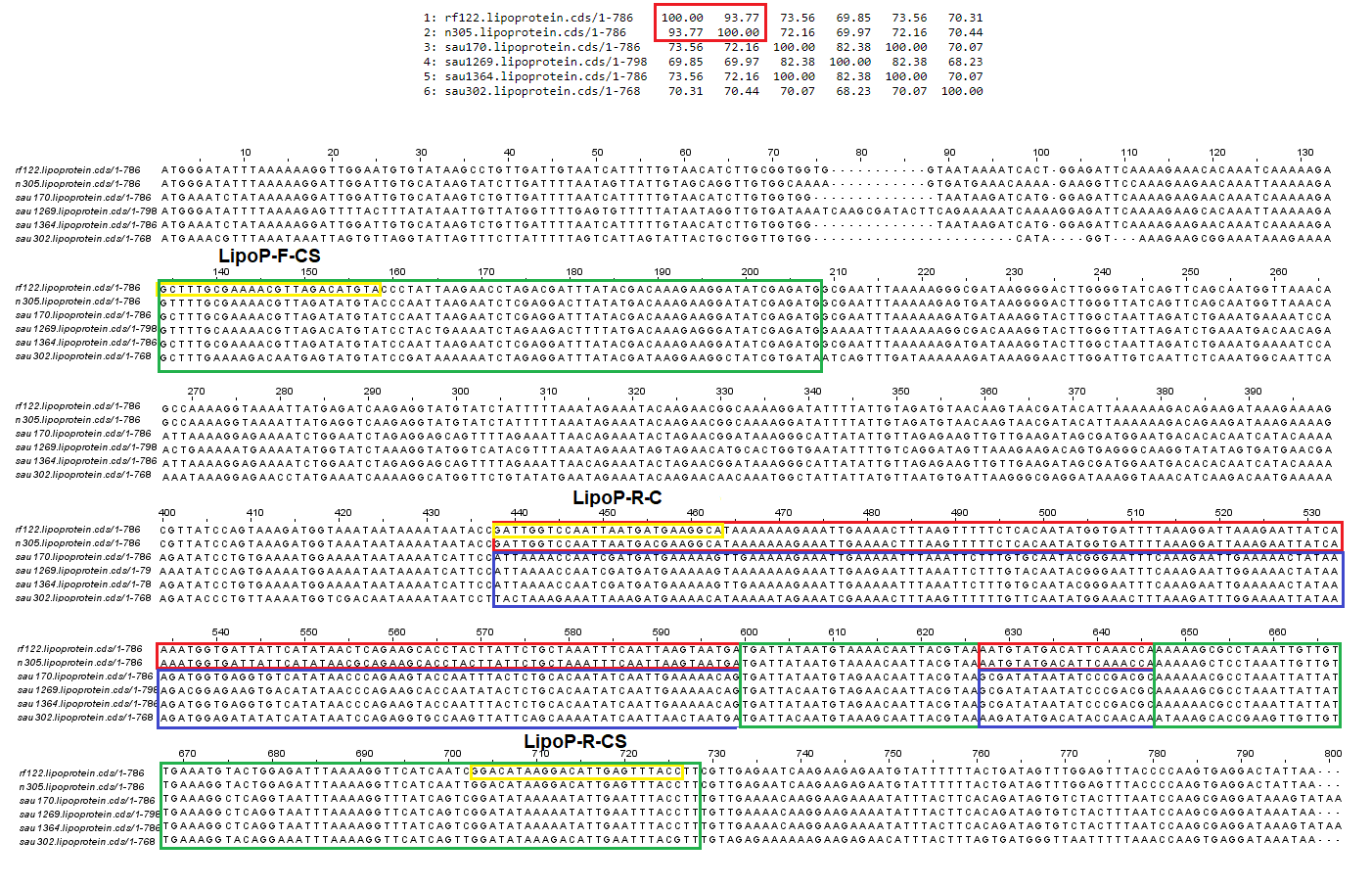

Supplement: S3 Fig — At the top, the percent identity matrix created with Clustal 2.1 of the lipoprotein, built with the amino acid sequence for the genomes of S. aureus strains Sau170, Sau302, Sau1269, Sau1364, RF122, and N305. The region conserved in RF122 and N305 is boxed in red. At the bottom, a multiple sequence alignment of the lipoprotein, also created with Clustal Omega, and the design of the sets of primers LipoP-F-CS/LipoP-R-C and LipoP-F-CS/LipoP-R-CS over the regions conserved among the clinical and subclinical strains (LipoP-F-CS and LipoP-R-CS) and over the region conserved between the clinical strains only (LipoP-R-C), all boxed in yellow. The red boxes correspond to the regions conserved only in the clinical strains, while the blue boxes correspond to the regions conserved in the subclinical strains. Highly conserved regions are boxed in green. (TIF) [file pone.0220804.s003.tif]

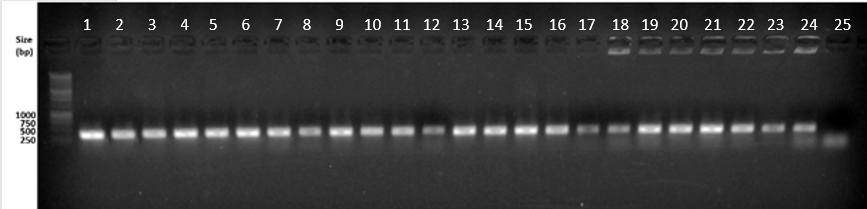

Supplement: S4 Fig — The PCR reaction was performed with the DNA extracted from the isolates S. aureus 170 (1), 302 (2), 1269 (3), 1364 (4), 308 (5), 340 (6), 403 (7), 1001 (8), 1311 (9), 1315 (10), 1323 (11), strain ATCC 29213 (12) and the DNA extracted from the isolates S. aureus 76 (13), 216 (14), 1439 (15), 3909 (16), 2555 (17), 5T18-19 (18), 9T18-16 (19), 10T18-59 (20), 10T18-68 (21), 14T18-13 (22), 22T18-52 (23), 22T17-54 (24). Water was used as a negative control (25). Promega 1 kb DNA Ladder was used as a molecular weight marker. (TIFF) [file pone.0220804.s004.tiff]

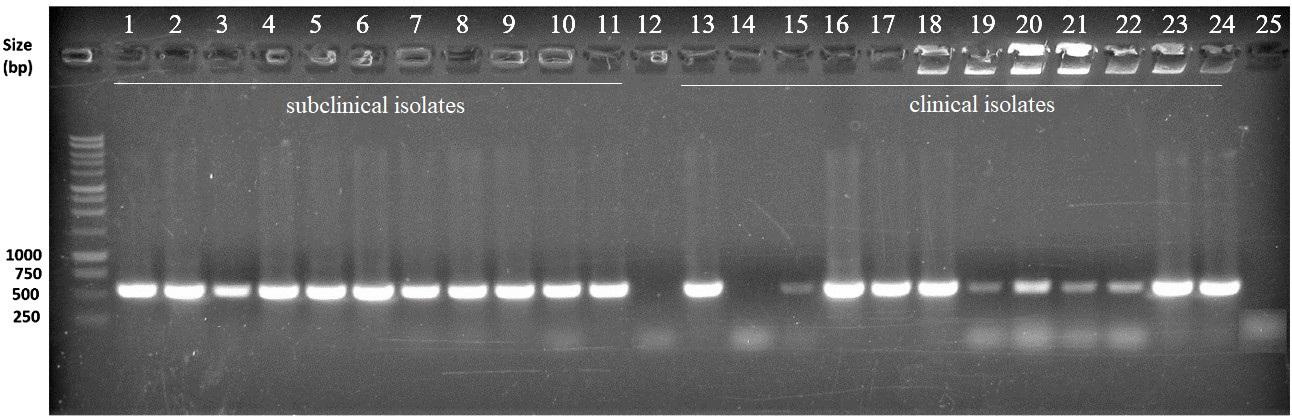

Supplement: S5 Fig — The PCR reaction was performed with total DNA extracted from subclinical mastitis isolates S. aureus 170 (1), 302 (2), 1269 (3), 1364 (4), 308 (5), 340 (6), 403 (7), 1001 (8), 1311 (9), 1315 (10), 1323 (11) and from the clinical mastitis isolates S. aureus 76 (13), 216 (14), 1439 (15), 3909 (16), 2555 (17), 5T18-19 (18), 9T18-16 (19), 10T18-59 (20), 10T18-68 (21), 14T18-13 (22), 22T18-52 (23), 22T17-54 (24). Total DNA from S. aureus ATCC 29213 (12). Water was used as a negative control (25). Promega 1 kb DNA Ladder was used as a molecular weight marker. (TIFF) [file pone.0220804.s005.tiff]

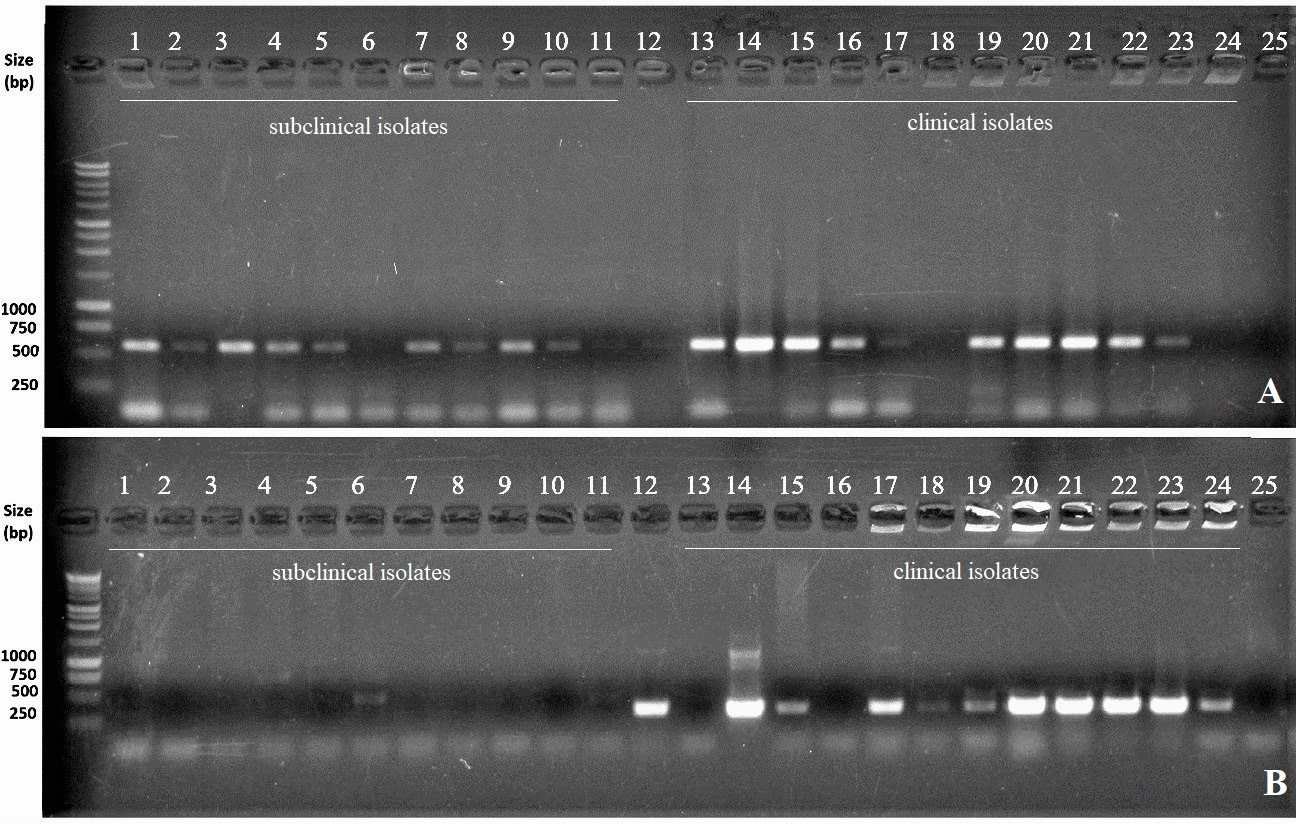

Supplement: S6 Fig — At the top (A), the primers LipoP-F-CS and LipoP-R-CS were used in a PCR reaction lewith DNA extracted from the subclinical mastitis isolates S. aureus 170 (1), 302 (2), 1269 (3), 1364 (4), 308 (5), 340 (6), 403 (7), 1001 (8), 1311 (9), 1315 (10), 1323 (11) and from the clinical mastits isolates S. aureus 76 (13), 216 (14), 1439 (15), 3909 (16), 2555 (17), 5T18-19 (18), 9T18-16 (19), 10T18-59 (20), 10T18-68 (21), 14T18-13 (22), 22T18-52 (23), 22T17-54 (24). At the bottom (B), the primers LipoP-F-CS and LipoP-R-C were used to amplify the same DNA Total DNA from S. aureus ATCC 29213 (12). Water was used as a negative control (25). Promega 1 kb DNA Ladder was used as a molecular weight marker. (TIFF) [file pone.0220804.s006.tiff]
